# Primary Transpupillary Thermotherapy of Choroidal Melanocytic Lesions

**DOI:** 10.4103/0974-9233.80711

**Published:** 2011

**Authors:** Kaan Gündüz, Melisa Zişan Karslioğlu, Kenan Köse

**Affiliations:** Department of Ophthalmology, Ankara University Faculty of Medicine, Ankara, Turkey; 1 Department of Biostatistics, Ankara University Faculty of Medicine, Ankara, Turkey

**Keywords:** Branch Retinal Vein Occlusion, Choroidal Melanoma, Choroidal Nevus, Cystoid Macular Edema, Macular Pucker, Neovascular Glaucoma, Pigment Dispersion into the Vitreous Cavity, Retinal Neovascularization, Ruthenium-106 Plaque Radiotherapy, Transpupillary Thermotherapy

## Abstract

**Purpose:**

To evaluate the role of primary transpupillary thermotherapy (TTT) in the treatment of choroidal melanocytic lesions.

**Materials and Methods:**

Retrospective chart review of 24 patients (24 eyes) with choroidal melanocytic lesions, including 20 choroidal melanoma and four choroidal nevus treated with primary TTT. Choroidal nevus cases treated with primary TTT either demonstrated risk factors for growth into an early melanoma or had overlying choroidal neovascularization.

**Results:**

The mean initial tumor basal diameter was 6.6 (3.0-10.0) mm and the mean initial tumor thickness was 3.0 (1.0-5.0) mm. The mean number of TTT sessions was 2.5 (1-6). The mean decrease in tumor thickness was 1.2 mm (from 3.0 to 1.8 mm) at a mean follow-up of 22.7 (range 3-90) months. On the LogMar scale, visual acuity was stable at 1.0. Complications occurred in 50% of eyes. The most frequent complications were vitreous hemorrhage [5 patients (20.8%)], focal cataract [5 patients (20.8%)], iris atrophy [4 patients (16.6%)] and posterior synechia [4 patients (16.6%)]. There was no significant difference in the complication rate with respect to tumor thickness >3 mm versus tumor thickness ≤3 mm and juxtapapillary versus nonjuxtapapillary location (Fisher’s exact test, P>0.05). Kaplan-Meier curves showed that 9% of eyes develop recurrence by 1 year and 27% develop recurrence by 5 years after primary TTT. Two eyes (8.3%) were enucleated because of neovascular glaucoma and one eye (4.1%) was exenterated because of extraocular tumor recurrence. Globe salvage was achieved in 21 patients (87.5%). One patient (4.1%) with extraocular tumor recurrence developed liver metastasis and expired.

**Conclusions:**

Although TTT may be useful in the treatment of small choroidal melanocytic lesions, the high complication and recurrence rates warrant close monitoring of patients after primary TTT even when a flat chorioretinal scar has been achieved.

## INTRODUCTION

Transpupillary thermotherapy (TTT) was initially developed as an adjunctive treatment to plaque radiotherapy by Oosterhuis *et al*.^1^ Experimental studies showed that thermal penetration of the tumor reaches a maximum depth of 4 mm, thereby making TTT a potentially ideal treatment for small choroidal melanomas.^2^ Primary TTT has recently gained popularity as an outpatient procedure for the treatment of small choroidal melanomas, especially for tumors near the critical visual areas, where radiation would potentially result in vision loss.^3^,^4^ TTT has a number of advantages, including lower cost than plaque radiotherapy, avoidance of invasive surgery and of radiation complications, and convenience of outpatient treatment. TTT is done with a widely available infrared diode laser system.

Despite the initial enthusiasm for TTT, analyses of long-term patient data showed that primary TTT was associated with high rates of complications and recurrence.^5^–^9^ Therefore, the role of TTT as primary treatment for choroidal melanomas has been questioned.

During a 10-year period from 1999 to 2009, we used primary TTT in 24 eyes with choroidal melanocytic lesions. Herein, we report the results of TTT in this small cohort.

## MATERIALS AND METHODS

We reviewed the clinical charts of 24 patients who had been treated with primary TTT for choroidal melanocytic lesions (choroidal melanoma and choroidal nevus) in one eye on the Ocular Oncology Service, Ankara University Faculty of Medicine. An Institutional Review Board (IRB) approval was obtained for reviewing and publishing the results. The eligibility criteria for this study included eyes with choroidal melanocytic lesions <10 mm in base diameter and <5 mm in thickness, located within 5 mm of the optic disc and fovea. These were patients who were not condidates for plaque radiotherapy surgery because of medical contraindications or refusal to have invasive surgery, and patients with small tumors near critical visual areas.

Each patient underwent complete ophthalmic examination, including visual acuity testing (measured in Snellen and converted into LogMar for purposes of this study), intraocular pressure measurement, biomicroscopy, indirect ophthalmoscopy, A and B scan ultrasonography and fluorescein angiography. Liver function tests, chest radiography and abdominal ultrasonography were performed in all patients for systemic metastasis evaluation.

Treatment indications for choroidal nevi were two-fold: 1) presence of two out of five risk factors for growth including orange pigment (lipofuscin), subretinal fluid, juxtapapillary location, thickness >2 mm and symptoms; and 2) presence of choroidal neovascularization over a subfoveal or juxtafoveal nevus.

After retrobulbar block, TTT was applied via a fundus contact lens, using infrared diode laser emitting at 810 nm attached to a slit-lamp biomicroscope. The spot size was set at 3.0 mm. Each spot was kept on the tumor surface for 1 minute. The tumor surface was covered with spots overlapping with each other by one-third of the spot width. The energy level was started at 300 mW and increased until the surface of the tumor developed a light grayish discoloration and opacification, with treatment ending before a whitish reaction occurred on the tumor surface. TTT was repeated at 3-month intervals, depending on the regression pattern of the tumor. Success of treatment was defined as a flat chorioretinal scar or a stable, regressed tumor with less than 2 mm thickness on ultrasonography.

Patients were followed up at 3, 6, 9 and 12-month intervals during the first year and at 6-month intervals thereafter once tumor regression had been achieved. At each follow-up visit, complete ocular examination and A and B mode ultrasonography were performed. Fluorescein angiographic studies were repeated as necessary. Patients developing marginal recurrence during follow-up were treated with additional TTT. Further treatments, including enucleation and exenteration, were performed, as required.

Statistical analysis of the complication rate with respect to tumor thickness >3 mm versus tumor thickness ≤3 mm, and juxtapapillary versus nonjuxtapapillary location were carried out using Fisher’s exact test. Kaplan-Meier curves were plotted to estimate the risk of recurrence over time.

## RESULTS

Twenty-four eyes of 24 patients with choroidal melanocytic lesions were treated with primary TTT between March 1999 and November 2009. Twelve patients (50%) were males and twelve (50%) were female. Five (20.8%) patients had diabetes mellitus, four (16.6%) had hypertension, and four (16.6%) had atherosclerotic cardiovascular disease. Seventeen (70.8%) patients had no systemic problems. Twenty eyes (83.3%) had choroidal melanomas and four eyes (16.6%) had choroidal nevi. One of 20 eyes had diffuse choroidal melanoma. Twenty-one tumors were pigmented, two were partially pigmented and one tumor was amelanotic. Six eyes had juxtapapillary tumors. Results are summarized in Table 1.

**Table 1:** Tumor features, complications, recurrence, eye status and follow-up data in 24 patients treated with primary transpupillary thermotherapy

Tumor type/ location	Thickness (mm) Initial/Final	Base (mm)	Color	Number of TTT sessions	Complications	Recurrence	Enucleation/ Exenteration	Visual acuity (logMar) Initial/ Final	Follow-up (Months)
CM/JP	2.0/0	3.0	P	2	BRVO, VH IA, FC, PS	-	-	1.0/0	90
CN/NJP	2.0/1.0	5.0	P	1	-	-	-	0.5/0.3	27
CM/JP	2.0/2.0	6.0	P	2	IA	IO	-	1.0/1.6	37
CN/NJP	1.5/1.0	7.0	P	1	-	-	-	1.6/1.6	3
CM/NJP	4.0/1.0	6.0	P	3	PD	-	-	0.3/1.6	50
CM/NJP	2.3/1.2	6.0	PP	4	ERM, IA, FC, PS	-	-	1.0/1.6	30
CM/JP	3.8/3.5	9.0	P	2	VH, RD, FC	-	-	0.1/3.0	18
CM/JP	5.0/5.0	10.0	P	2	-	-	-	1.6/1.9	3
CM/JP	3.0/2.0	6.0	P	2	FC, PS, NVG, VH	-	Enucleated	1.9/3.0	10
CM/NJP	4.5/2.0	4.0	P	3	VH, PD	-	-	1.6/1.0	16
CN/NJP	1.6/1.6	4.5	P	1	-	-	-	0.1/0.1	3
CM/NJP	4.0/2.0	9.0	P	2	-	-	-	1.6/0.8	6
CM/NJP	3.0/2.0	6.0	P	3	-	-	-	0/0	6
CM/NJP	5.0/0	7.5	P	3	-	-	-	1.6/1.6	6
CM/NJP	2.5/0	6.0	P	2	ERM	-	-	0.5/0	68
CM/NJP	1.8/0.9	3.0	P	2	-	-	-	0.2/0	9
CM/NJP	3.6/2.5	6.0	P	3	-	-	-	0.6/0.5	6
CM/NJP	4.5/4.5	10.0	AM	2	PD	-	-	1.9/1.9	3
CM/NJP	2.0/0	7.5	P	6	BRVO, IA	IO, EO	Exenterated	1.6/1.9	54 (expired)
CN/NJP	1.0/3.8	6.0	P	2	ERM	IO	-	0.6/1.9	77
CM/NJP	2.5/2.5	5.0	P	1	-	-	-	1.9/1.9	3
CM/JP	5.0/0	7.0	PP	3	FC, PS, BRVO, NVG, VH	-	Enucleated	1.6/1.9	20
CM/NJP	2.0/1.5	3.0	P	1	-	-	-	1.6/1.6	3
CM/NJP	2.5/2.5	7.0	P	1	-	-	-	1.0/1.0	3

CM: Choroidal melanoma, CN: Choroidal nevus, JP: Juxtapapillary, NJP: Non-juxtapapillary, P: Pigmented, PP: Partially pigmented, AM: Amelanotic, BRVO: Branch retinal vein occlusion, IA: Iris atrophy, FC: Focal cataract, VH: Vitreous hemorrhage, PD: Pigment dispersion into the vitreous cavity, ERM: Epiretinal membrane, PS: Posterior synechia, NVG: Neovascular glaucoma, RD: Retinal detachment, IO: Intraocular, EO: Extraocular, (-): Denotes absence of the specified event, TTT: Transpupillary thermotherapy

The mean patient age was 56.9 years (range 34-82 years). The initial visual acuity ranged from counting fingers at 20 cm to 20/20. The mean initial visual acuity was 1.0 logMAR. The mean largest tumor base diameter was 6.6 mm (range 3.0-10.0 mm) and the mean tumor thickness was 3.0 mm (range 1.0-5.0 mm). The mean distance to the optic disc was 1.5 mm (range 0-6.0 mm) and the mean distance to the foveola was 0.9 mm (range 0-4.0 mm). Of 24 patients, 20 (80%) had symptoms, 19 (79%) had subretinal fluid, 19 (79%) had orange pigment and 17 (70%) had drusen initially. One eye with a nonjuxtapapillary choroidal nevus had overlying choroidal neovascular membrane. The mean number of TTT sessions was 2.3, ranging from 1 to 6. The mean powers used were as follows: session 1, 565.9 mW (range 400-800 mW); session 2, 577.6 mW (range 450-800 mW); session 3, 590 mW (range 500-820 mW); session 4, 725 mW (range 600-850 mW); session 5, 600 mW; and session 6, 600 mW. The spot size was 3 mm. The mean TTT time for each application session was 8.5 minutes.

**Figure 1:**
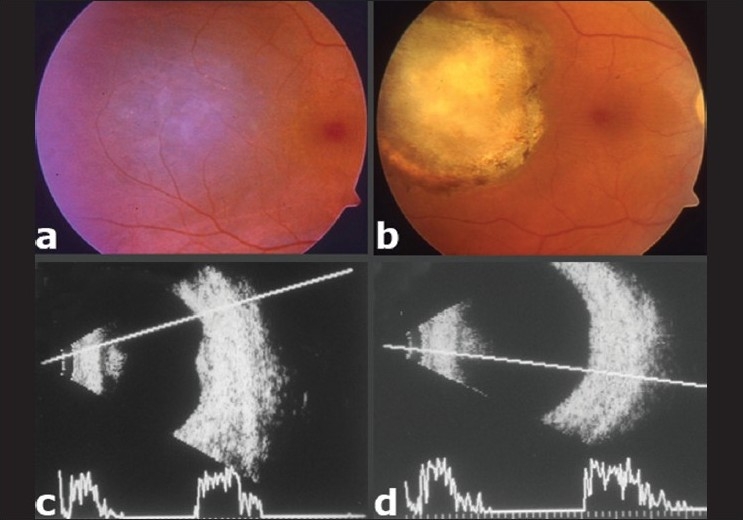
(a) Pigmented choroidal melanoma located temporal to the fovea. (b) Complete regression of the melanoma into a flat chorioretinal scar after three sessions of transpupillary thermotherapy. (c) B-mode ultrasonogram shows a 2.5 mm-thick choroidal melanoma with acoustic solidity at baseline. The vitreous appears clear and there is no extraocular extension. (d) B-mode ultrasonogram after three sessions of transpupillary thermotherapy demonstrates complete regression of choroidal melanoma

**Figure 2:**
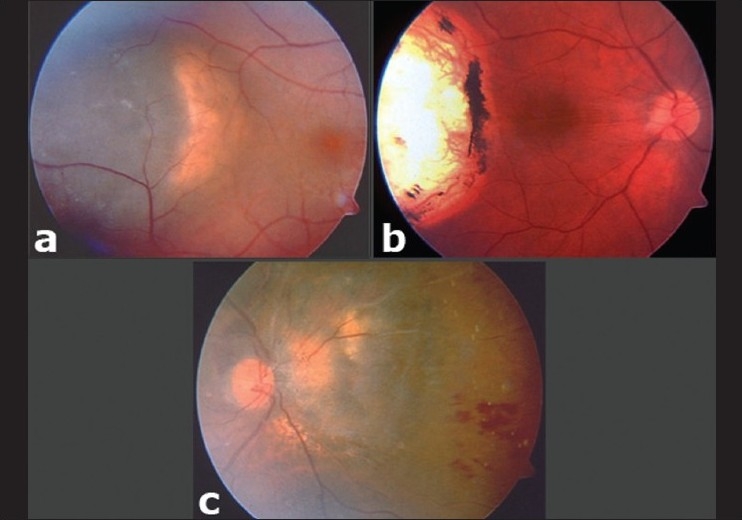
(a) Partially pigmented choroidal melanoma located temporal to the fovea. (b) Complete regression of the melanoma into a flat chorioretinal scar after three sessions of transpupillary thermotherapy with an epiretinal membrane in the macular area extending from the scar base to the optic disc. (c) Branch retinal vein occlusion, which developed after one session of transpupillary thermotherapy in an eye with juxtapapillary melanoma located nasal to the optic disc

**Figure 3:**
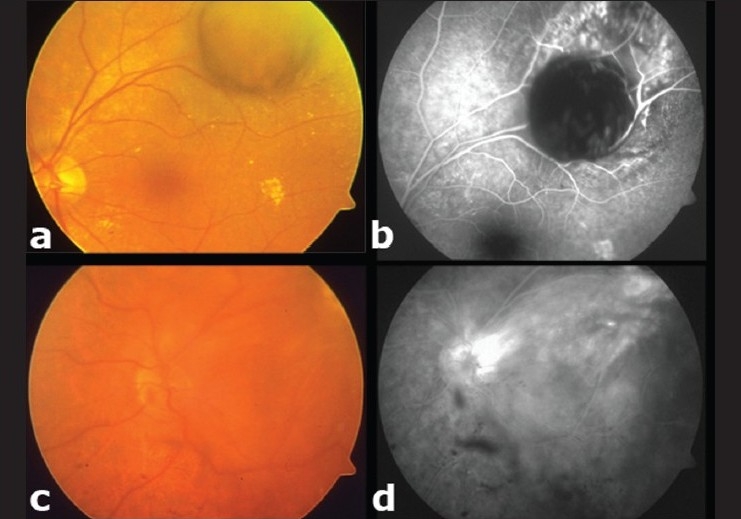
(a) Mushroom-shaped choroidal melanoma 4.5 mm in thickness located superotemporally posterior to the equator of the left eye. (b) Mid-phase fluorescein angiogram shows hypofluorescence in the lesion resulting from retinal invasion of the choroidal melanoma. (c) After three sessions of transpupillary thermotherapy, the tumor demonstrated significant regression with intravitreal pigment dispersion. (d) Mid-phase fluorescein angiogram shows hypofluorescence resulting from intravitreal pigment deposits

The mean follow-up time was 22.7 months (range 3-90 months). The mean decrease in tumor thickness was 1.2 mm (from 3.0 to 1.8 mm; range 0-6.8 mm) by the end of the follow-up. Five eyes had flat chorioretinal scars after TTT [Figure 1a–d]. Final visual acuity ranged from hand motions to 20/20. The final mean visual acuity was stable at 1.0 LogMar compared to baseline. Twelve eyes (50%) had no complications and 12 (50%) had one or more complications. The complications were vitreous hemorrhage in five eyes (20.8%), focal cataract in five eyes (20.8%), posterior synechia in four eyes (16.6%), focal iris atrophy in four eyes (16.6%), epiretinal membrane in three eyes (12.5%; Figure 2a, b), branch retinal vein occlusion (BRVO) in three eyes (12.5%;  Figure 2c), pigment dispersion into the vitreous cavity in three eyes (12.5%; Figure 3a–d), neovascular glaucoma in two eyes (8.3%) and retinal detachment/proliferative vitreoretinopathy (PVR) in one eye (4.1%). Vitreous hemorrhage developed secondary to BRVO or neovascular glaucoma in three eyes, retinal detachment and PVR in one eye and tumor necrosis in one eye with tumor thickness of 4.5 mm. Pigment dispersion into the vitreous cavity was seen in eyes with mushroom-shaped choroidal melanoma with retinal invasion. No retinal breaks could be identified in the eye with retinal detachment and PVR.

We evaluated the complication rate with respect to tumor color, location and thickness. Owing to the small number of amelanotic and partially pigmented tumors, it was not possible to perform statistical analysis of complication rate with respect to tumor color. Five of six eyes with juxtapapillary tumors and seven of 18 eyes with nonjuxtapapillary tumors developed complications. Fisher’s exact test indicated no difference in the complication rate between juxtapapillary and nonjuxtapapillary tumors, although juxtapapillary tumors had a numerically higher rate of complications (*P*=0.077). Of 15 tumors with thickness <3 mm, six (40%) developed one or more complications, while nine (60%) had no complications. Of nine tumors with thickness ≥3 mm, 5 (55.6%) developed one or more complications, while four (44.4%) had no complications. Fisher’s exact test indicated no difference in the complication rate between tumors >3 mm in thickness versus tumors ≤3 mm in thickness (*P*=0.375).

**Figure 4:**
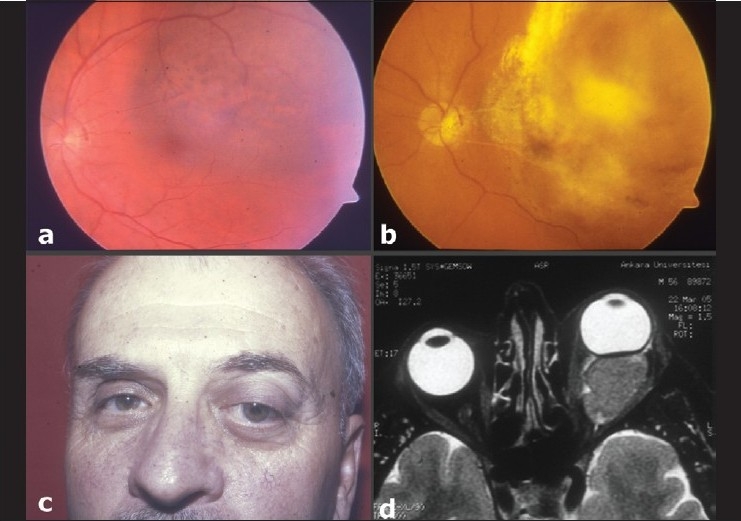
(a) Diffuse macular melanoma at baseline. (b) The patient underwent three sessions of transpupillary thermotherapy at 3-month intervals initially but the tumor recurred. Subseqently, he underwent Ru-106 plaque radiotherapy and two additional sessions of transpupillary thermotherapy, with regression of the intraocular recurrent tumor to a flat intraocular scar. There is retinal vascular obliteration from transpupillary thermotherapy. (c) At 48 month follow-up, thirty months after the diagnosis of intraocular recurrence, the patient presented with proptosis of the affected left eye. (d) T2-weighted orbital MRI shows a lesion isointense with respect to the cerebral gray matter and extraocular muscles in the left orbit. The patient underwent lid-sparing exenteration and histopathological examination demonstrated epithelioid cell melanoma in the orbit

**Figure 5:**
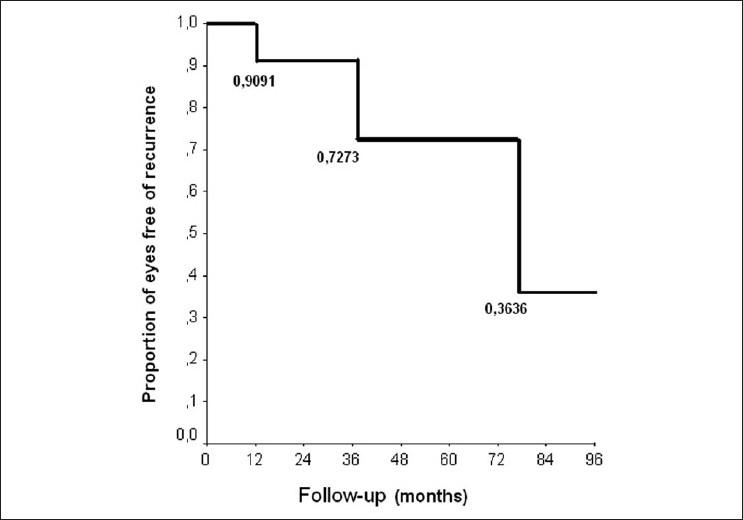
Kaplan-Meier curve shows the proportion of patients free of recurrence (about 90% at 1 year and about 73% at 5 years after initial diagnosis)

Three eyes developed marginal tumor recurrence at 12 months (the eye with diffuse melanoma), 37 months and 77 months [Figure 4a–d]. These recurrences were treated successfully with additional TTT in two eyes and with combined TTT and Ruthenium-106 plaque radiotherapy in one eye (eye with diffuse melanoma). The eye with diffuse melanoma, despite a flat chorioretinal scar intraocularly, developed extraocular tumor growth at 48-months follow-up. Using Kaplan-Meier curves, the proportion of patients free of recurrence was 91% of eyes at 12 months and 73% at 60 months (95% confidence interval; Figure 5).

Two eyes with juxtapapillary choroidal melanomas underwent enucleation for intractable neovascular glaucoma and vitreous hemorrhage resulting from retinovitreal neovascularization. The eye with extraocular tumor recurrence was exenterated. Overall, the globe salvage rate was 21/24 (87.5%). One patient with extraocular tumor growth died of metastatic melanoma at 54 month follow-up.

## DISCUSSION

In our study, we treated choroidal melanomas and choroidal nevi demonstrating an at least 50% risk of growth into a melanoma. These choroidal nevi demonstrated two or more of five risk factors for growth including orange pigment, subretinal fluid, thickness >2 mm, juxtapapillary location and symptoms or had overlying choroidal neovascularization. Treatment of borderline choroidal nevi with suspicious features suggestive of transformation into an early melanoma is generally recommended.^10^

TTT produces temperatures of 45-60°C, causing immediate direct cytotoxic effect, cell damage and tumor necrosis.^2^ Tumor regression starts within weeks and continues for months, resulting in a regressed tumor or an atrophic chorioretinal scar at the site of the tumor. Tumor regression usually takes place faster and in a more profound fashion compared to various radiotherapeutic methods.

TTT may be associated with several complications, including macular pucker and retinal traction, refractory cystoid macular edema, branch retinal vein and artery occlusions, focal iris atrophy, increased sensory retinal detachment and pigment dispersion into the vitreous cavity.^5^–^9^ Aaberg *et al*. reported complications in about one-third of eyes (43 of 135 eyes) that underwent under primary TTT.^8^ Complications in the series of Aaberg *et al*. included macular pucker in 15 eyes (11%), vascular occlusions in 13 eyes (10%), refractory cystoid macular edema in 7 eyes (5%), macular hole in 2 eyes (1%), vitreous hemorrhage in 2 eyes (1%), rhegmatogenous retinal detachment in 1 eye (<1%) and exudative retinal detachment in 1 eye (<1%).^8^ Shields *et al*. reported that in their study of 256 eyes that underwent TTT, the most common complications were retinal traction in 112 eyes (44%), BRVO in 104 (41%) eyes, branch retinal artery occlusion in 31 (12%) eyes, cystoid macular edema in 24 eyes (9%) and retinal neovascularization in 16 eyes (6%).^5^ In our series, we noted relatively high frequencies of vitreous hemorrhage (21%), focal cataract (21%), iris atrophy (17%) and posterior synechia (17%). Focal cataract, iris atrophy and posterior synechia can be caused by inadvertent touching of the mid-dilated pupil by the broad laser beam. Therefore, it is important to keep the pupils fully dilated throughout the procedure. Should the pupil become miotic, the TTT application should be stopped and pupillary dilation should be re-established. We observed intravitreal pigment dispersion in three eyes in our study, a complication that has not been described in the two large series on the subject.^5^,^8^ We suspect that eyes with mushroom-shaped tumors and retinal invasion are prone to tumor necrosis and subsequent pigment release into the vitreous cavity because the circulation to the tumor apex is strangulated by the break in Bruch’s membrane, leading to less dissipation of heat after TTT.

One of the most worrisome aspects of primary TTT is the high recurrence rate. Using Kaplan-Meier curves, two groups independently found that approximately 20% of patients (19%^8^ and 22%^5^) treated with TTT develop treatment failure by 5-year follow-up. The highest recurrence rates (55% at 5 years) were reported by Pan *et al*.^7^ and Spire *et al*.^9^ The mean time to the development of recurrence after primary TTT was 22 months in two series.^5^,^6^ Aaberg *et al*.^8^ reported that intraocular tumor recurrence developed in seven eyes (5%) and extraocular recurrence in four eyes (3%) out of 135 eyes treated with primary TTT. Univariate analysis determined that tumor diameter > 10 mm, tumor thickness > 3 mm and tumors exhibiting high-risk characteristics (subretinal fluid, orange pigment and thickness > 2 mm) were significant predictors of failure in Aaberg et al’s series. Patient age, gender, number of treatments and proximity of the tumor to the disc or fovea were not predictive of failure.^8^ Treatment failure occurred as late as 99 months.^8^ Shields *et al*.^5^ found that risk factors for recurrence after TTT were increasing number of treatment sessions and tumor location abutting the optic nerve head. We found a recurrence rate of 27% by 5 years using Kaplan-Meier curves in our study.

Zaldivar *et al*.^11^ reviewed the clinicopathological features of eyes enucleated after failed primary TTT and found that intrascleral extension of the tumor is the main reason for the failure of primary TTT. In fact, the Collaborative Ocular Melanoma Study found that intrascleral melanoma cells were present in 56% of the enucleated eyes with choroidal melanoma.^12^ Melanoma cells in the nonpigmented sclera remain unaffected by the heat effects of TTT and may grow into the eye or orbit later as clinically evident tumor recurrence.^11^ Even completely regressed flat choroidal melanomas after primary TTT can demonstrate extraocular extension on ultrasonography, as was the case in one of our patients. Therefore, patients undergoing primary TTT need to be followed up indefinitely with both clinical and ultrasonographic examinations.

Enucleation after primary TTT is usually done for tumor recurrence or intractable treatment complications. Shields *et al*.^5^ reported that enucleation was performed in three of 256 eyes (1%) due to nonresponsive amelanotic choroidal melanomas. Aaberg *et al*. reported an enucleation rate of 8.8% (12 of 135 eyes) for recurrent tumor.^8^ In our series, two eyes underwent enucleation for neovascular glaucoma and another eye underwent exenteration for extraocular tumor recurrence, representing a globe loss rate of 12.5%.

TTT usually achieves preservation of vision, as in our series. However, final vision largely depends on the location of the tumor and the complications arising after treatment. Vision is maintained or improved in about half of the patients treated with primary TTT and is reduced or lost in the other half.

Shields *et al*.^5^ reported that distant metastasis to liver was seen in two of 256 patients (0.8%) at 24 months and 39 months after TTT, respectively. Aaberg *et al*.^8^ found a melanoma-specific mortality rate of 3% at a mean of 5-year follow-up. One of 24 patients (4.1%) developed liver metastasis after extraocular tumor recurrence in our series. This relatively low mortality rate is atttributable to the small dimensions of the choroidal melanomas at baseline.

Our study has certain limitations. There was no difference in the complication rate according to a juxtapapillary versus nonjuxtapapillary location and tumor thickness >3 mm versus tumor thickness ≤3 mm in our study. These findings are probably explained by the low number of cases analyzed that failed to reach statistical significance. The novel features of our study included TTT of choroidal melanoma >4 mm and <5 mm in thickness, treatment of suspicious choroidal nevi with borderline features suggestive of transformation into an early melanoma, and treatment of choroidal nevus with overlying choroidal neovascularization. Although primary TTT is usually recommended for tumors with a thickness <4 mm, there were five cases with tumor thickness >4 mm and <5 mm treated with primary TTT in our series.^5^ These patients were unable to undergo brachytherapy surgery and TTT was the only therapeutic option available. A review of the published papers on the subject reveals that there were occasional melanoma cases up to 6.6 mm in thickness treated with primary TTT.^3^,^5^,^8^ TTT is also a therapeutic option in cases of choroidal neovascularization overlying a choroidal nevus, as demonstrated by one of the cases in our series.

In conclusion, TTT may have a limited role in the primary treatment of small choroidal melanocytic lesions, especially near the visually critical areas. About 30-50% of the eyes develop complications, and these complications need to be followed carefully, as they can lead to vision loss and even globe loss. It should be borne in mind that about 25% of the eyes fail TTT by 5-year follow-up. Therefore, patients treated with TTT should be followed with both clinical examination and ultrasonography to detect intraocular and extraocular tumor growth, even when a flat chorioretinal scar has been achieved.
